# Advancing a Digital Health Ethics Framework for AD/ADRD

**DOI:** 10.1002/alz.090843

**Published:** 2025-01-09

**Authors:** Emily A. Largent, Jason Karlawish, Anna Wexler

**Affiliations:** ^1^ University of Pennsylvania, Philadelphia, PA USA

## Abstract

**Background:**

“Digital health” is a broad term that encompasses a heterogeneous set of scientific concepts and technologies. While digital health tools have the potential to contribute to better health and health care for individuals and communities, they also pose ethical challenges – particularly in the context of Alzheimer’s Disease and Alzheimer’s Disease and Related Dementias (AD/ADRD). There is no single established ethical framework, however, to aid individuals in evaluating the ethical dimensions of digital health tools in the context of AD/ADRD. To advance the development of an ethical framework, we draw on our experiences with the Penn Artificial Intelligence and Technology (PennAITech) Collaboratory for Healthy Aging, which assists in the identification, development, and dissemination of innovative technologies and artificial intelligence methods to support older adults, including those with AD/ADRD in their home environment.

**Methods:**

We employed two primary methods to assist with the development of our framework. First, we reflected on consultations with product developers that we conducted in our roles as leads of the PennAITech Ethics and Policy Core. The Core seeks to identify and address real‐world ethical, regulatory, and policy issues that arise at the intersection of aging, AI, and digital health through consultations. Second, we reviewed the relevant clinical, scientific, ethics, law, and policy literature to identify and characterize enduring and emerging issues.

**Results:**

We will present our framework for digital health ethics in the context of AD/ADRD and use real world examples to identify and characterize a core set of ethical considerations that cut across many types of digital health tools. These include: assessing and addressing bias; considering risk‐benefit tradeoffs; protecting user privacy; considering environmental impact; measuring value along technical, clinical, usability, and cost dimensions; educating users; and promoting equitable access. For each issue, we also identify potential means of addressing these challenges.

**Conclusion:**

Widespread adoption of digital health tools has the potential to improve health and health care for individuals, caregivers, and communities affected by AD/ADRD, but realizing this potential requires anticipating and addressing ethical challenges.

